# Reduction but not elimination: health inequalities among urban, migrant, and rural children in China—the moderating effect of the fathers’ education level

**DOI:** 10.1186/s12889-019-7522-6

**Published:** 2019-09-04

**Authors:** Dianxi Wang

**Affiliations:** Institute of Evidence Law and Forensic Science, China University of Political Science and Law, No. 25, Xitucheng Road, Haidian District, Beijing, Zip Code:100088 China

**Keywords:** Health inequalities in children, Urban children, Migrant children, Rural children, Fathers’ education level

## Abstract

**Background:**

Given the urban-rural structure and the increase in rural-to-urban migration, three types of children have emerged in contemporary China: rural, urban, and migrant children. Health disparities among these types of children have caused widespread concern, being the main contributor to health inequalities among children in China. The purpose of this study was to investigate health disparities among these children and the mechanisms underlining them.

**Methods:**

This research applied multiple linear regression to data obtained from the Chinese Education Panel Survey (CEPS), a national representative survey of 7772 students from 2014 to 2015. Multiple linear regression with interactive terms was used to explore how gender and father’s education moderate the degree of health inequalities among the children. ‘Height for age Z-scores’ (HAZ) was deployed as the indicator of the children’s health status, with larger scores indicating better health status.

**Results:**

The findings of the current study were threefold: First, this study found significant health disparities among the three types of children. Urban children are generally the healthiest (M = 1.064), followed by migrant children, (M = 0.779) and rural children (M = 0.612). Second, fathers’ education significantly compensates for the heath disparities among the children. Fathers’ education has a larger effect in compensating a rural-migrant difference (b = − 0.018, *P* < 0.05) than a rural-urban difference (b = − 0.016, *P* < 0.1). Third, the compensating effect of the fathers’ education varies by gender. The compensating effect is larger for boys (b = 0.028, *P* < 0.001) than for girls (b = 0.025, *P* < 0.01).

**Conclusions:**

This study found significant health inequalities among urban, migrant, and rural children, which might be shaped by the distinction of urban-rural structure and the process of rural-to-urban migration in contemporary China. Fathers’ education also plays an important role in narrowing—but not eliminating—the health inequality between urban and rural children. Furthermore, the compensating effect of fathers’ education is higher for boys than for girls, reflecting the patriarchal tradition in China. The currents study suggests that to promote the healthy growth of children, it is necessary to consider the health inequalities among different types of children when developing health-related policies. Factors like family socioeconomic status and gender may likewise play an important role in the implementation of policies.

## Background

Since the beginning of reforms in China during the 1980s, the population has rampantly moved from rural to urban areas. The size of the migrant population has grown rapidly since the 1990s, from 21.35 million in 1990, to 244 million in 2017 [1]— an increase by nearly 12 times. More migrants have moved to the cities in search of better-paying jobs and more benefits; some parents choose to bring their children with them, while others have them remain in the countryside. In the context of the urban-rural division and the rural-to-urban migration in China, children are grouped according to their migration states: rural children, migrant children, and urban children. Rural children are those who were born in rural areas and did not migrate to urban areas. Although the size of rural child population dropped from 340 million in 1982, to 140 million in 2015, the number of rural children who were left behind by their parents reached a high of 61 million in 2015, accounting for 40% of all rural children and approximately 22% of all children in the country [2]. The second group, migrant children, are those whose Hukou (also referred to as ‘household registration’, which divides Chinese citizens into urban and rural residents—a distinction that carries social, economic, and political implications [3]) is not in their place of residence, and who have migrated with their parents and lived in the place of residence for more than half a year. Since the twenty-first century, the number of migrant children has proliferated: those under the age of 17 increased from 19.82 million in 2000 to 34.26 million in 2015 [2]. Lastly, urban children are those who are born in a major urban centre and do not migrate with their parents. The number of urban children in China was 68.08 million in 1982, but nearly doubled to 130 million by 2015 [2, 4]. As a derivative of population migration and rapid urbanization in China, both large-scale rural children and migrant children have attracted a great deal of academic attention, much of it focused on the quality of life and health of these children.

In recent years, scholars have found that the widening gap in health outcomes between populations is particularly evident among children [5]. As investigators extend health inequality research to children, health inequalities among children have become an important topic in both social science and public health research. Childhood health inequality is related to disparities in life course [6, 7], can affect the holistic process of individual growth and development, and is the beginning of the evolution of individual adult health. Therefore, studying children’s health inequalities has significant predictive effects for understanding adult health differences.

Existing research has identified significant differences in the health of different individuals during childhood using various indicators, such as infant mortality, child height, self-reported health, BMI, and a specific representation of children’s health inequalities, such as social inequalities in health, urban and rural differences in children’s health inequalities, and health inequalities between local and migrant children. For example, on the aspect of socioeconomic inequalities in health, some studies found that children of parents of a high socioeconomic status have better self-reported health than those of low socioeconomic status [8]. Based on data from 34 countries from 2002 to 2010, Elgar et al. found that the average BMI of school-aged children slightly increased and became more unequal between socioeconomic groups [9]. Currie et al. also found that there are persistent socioeconomic inequalities in self-reported health, psychosomatic symptoms, physical activity, and eating habits at both the individual and country level [10].

With regard to urban-rural differences in child health, data from China also shows that the Chinese Hukou System—the household registration management policy—exacerbates urban-rural differences in nutrition-related child health outcomes. The height z-scores of urban Hukou children are 0.25 higher than that of rural Hukou children. [11]. Some scholars studying sub-Saharan African countries found that despite urban-rural differences in children’s health inequalities, differences in children’s health within urban areas are greater than those between urban and rural areas. Furthermore, urban health advantages mask huge differences between urban poor and non-poor in sub-Saharan Africa [12].

In the field of children’s health inequalities, the health status of migrant children has been the focus of attention. The existing research has concentrated on the differences in the health indicators of migrant children and local children and confirms the health differences between migrant children and other children. For example, Xu and Xie found that migration has a significant positive impact on the children’s objective well-being [13], and there is no significant difference in health welfare between migrant children and local children. This indicated that the migration from rural to urban areas may be related to an improvement in the health status of children. As scholars have found, the risk of death for migrant children is similar or slightly higher than that of children still living in rural areas, and the chances of survival of migrant children born after the parents have settled in the city have been significantly improved [14, 15]. Additionally, scholars also found that the health indicators (preterm birth rate, birth weight, and infant mortality) of babies born to foreign-born women are better than those born to women born in the United States, stemming from the ‘selectivity’ of healthy women before conception [16]. The above findings are usually discussed under the framework of the healthy migration hypothesis, stating that those who are healthier are the ones who are more likely to migrate [17].

Given these health gaps, various scholars have focused on the health inequality mechanisms in children, mainly concentrating on socioeconomic inequalities in health. They believe that children’s health differences stem from unequal family socioeconomic status in early childhood, which reflects the impact of family origin on child health. Many have found that social and economic inequalities in children’s health are prevalent throughout the world [18–20], and this trend is consistent with uneven income distributions between the rich and the poor [9, 21]. Children from low socioeconomic groups exhibited poorer health performance, with children born to poor families having a lower level of health than children born to wealthy families [8, 22]. For example, some researchers showed that food shortages and low household income negatively influence health in preschool and school-age children in the United States [23]. Generally speaking, children from disadvantaged socioeconomic families start off living in poorer states of health and have less ability to benefit from economic and social progress [24]. In addition to physical health, there is a significant negative correlation between socioeconomic status and mental health in childhood: children who are disadvantaged socioeconomically are two to three times more likely to suffer from mental health problems [25]. Research also showed that disadvantaged social and economic status mainly cause children’s health inequality because these families have unequal access to medical resources and health care due to their social and economic conditions [26]. Poverty, as one of measurement indicators of a family’s socioeconomic situation, has been shown to have a negative impact on children’s health and development in many ways. Long-term poverty likewise has a negative impact on the health of children [27, 28]. In summary, the origins of social class are meaningfully associated with health inequalities in children, with significant healthy social gradient effects [29, 30].

In addition to family economic status, the influence of parents on their children’s health has also been the focus of attention in some literature. Many scholars found that the status of the mother has a significant impact on the health inequalities of children. For example, children of single mothers and those living alone have the highest mortality rates [31]. The mother’s education level is also a robust predictor for inequalities of child health and nutrition, and had positive correlation with long-term health outcomes of children [32, 33]. Scholars believe that the mother’s education has a significant effect on children’s health, affecting factors such as the mothers’ behaviour during pregnancy and influencing the use of health inputs [34]; mothers with high education levels have effective access to health knowledge and a strong ability to care for their children, have greater household income, better labour market participation, and wider maternal empowerment within the home [35]. Many studies also focus on the impact of fathers’ education on children’s health outcomes, and find that fathers’ education are positively related to self-rated health [36], with children of more highly-educated fathers having more advantageous health outcomes than their peers with less-educated fathers [37]. The effect of fathers’ education on infant and child mortality appears to be about one half that of mothers’ education [38]. However, some scholars believe that the association of fathers’ education with stunting could be confounded by mothers’ education [32]; thus, the independent effect of fathers’ education needs further verification. In other words, the existing research does not fully clarify the pathway of the influence of fathers’ education on children’s health, especially the mechanism of influence of fathers’ education on different types of children. Moreover, some scholars have pointed out that the degree of fathers’ education on children’s health varies substantially between East and West [36]. The traditional concept of ‘patriarchal system’ still has a profound impact, especially in China. The role, status, and responsibility of the father in the family is of vital importance and has a significant impact on the growth and development of the children. Therefore, this study mainly focuses on the impact of fathers’ education on the health of different types of children.

In sum, existing literature provides an in-depth analysis of health differences between urban and rural children and migrant and non-migrant children, and analyses the current situation and mechanisms of health inequalities in children, finding that children’s health inequality is a universal social phenomenon, and the difference in socioeconomic status is the main factor of health inequalities in children [9, 19, 39]. Scholars also found that population migration has caused structural differences in children’s health inequalities, and that parental migration can promote children’s chances of survival and cause real differentiation of migrant children and local children in health [13, 40]. However, existing research often analysed the health inequalities of migrant and non-migrant children, as well as those of urban and rural children in the context of urban-rural differences [11, 41], but paid less attention to the health differences or inequalities among the three groups of children discussed above—urban children, rural children, and migrant children. Comparing the health differences of the different groups of children is of great significance for understanding the internal mechanisms of children’s health inequalities. In addition, existing research tends to focus on the family socioeconomic status, especially the impact of the mother’s education level on children’s health inequalities [32, 37], and less on the impact of the fathers’ role on health differences of the above three types of children. Therefore, in order to fill the gaps in existing research and to further clarify the impact of fathers’ education on health inequalities in children, this study, based on CEPS data, investigates middle school students as the research object to analyse the health inequalities of urban children, rural children, and migrant children. This study investigates the effects of the fathers’ education level on the health inequalities of the three types of children, as well as the potential gender differences in the moderating effect of fathers’ education level. In this analysis, this research proposes the following three research hypotheses.

### Hypothesis 1

Urban children, migrant children, and rural children have significant differences in health levels. Urban children have higher levels of health than migrant children, and migrant children have higher levels of health than rural children.

### Hypothesis 2

The fathers’ education level has moderating effects on the health differences of the above three types of children. That is, the increase of the fathers’ years of education has different effects on the health of the three types of children.

### Hypothesis 3

The moderating effect of the fathers’ education level on the above three types of children’s health inequalities varies according to gender, and the moderating effect on male and female children is not the same.

## Method

### Data

The data used in this study comes from the China Education Panel Survey (CEPS, http://cnsda.ruc.edu.cn), a nationally representative, large-scale longitudinal survey project designed and implemented by the National Survey Research Centre at Renmin University of China, aimed at revealing the impact of family, school, community, and macro-social structure on individual educational outputs. The survey used middle school students as the survey object, applying a multistage probability proportionate to size sampling (PPS) method, and randomly selected 28 county-level units (counties, districts, and municipalities) from the national study as the survey points of the average level of education and proportion of the floating population. A total of 112 schools and 438 classes were selected from 28 county-level units for investigation. To date, the survey has been conducted in two waves: the baseline survey for the 2013–2014 school year, and the follow-up survey for the 2014–2015 school year. The current study mainly uses the data from the 2014–2015 school year survey, which had a response rate of 91.9%. After cleaning up the data, the final valid sample for this study was 7772 students.

### Measurement and variables

#### Dependent variable

According to the World Health Organization (WHO), the children’s health standard refers to the growth and development of children’s organs and tissues, psychological development, having a cheerful personality or optimistic mood, and strong adaptability to the environment [42]. In addition to indicators such as mortality and prevalence rates, nutritional status is an essential aspect of a child’s physical health. Anthropometric indicators (e.g., height, weight, etc.) are usually used to measure a child’s long-term health. The WHO recommends the ‘height for age Z-scores’ (HAZ) as a measurement indicator for comparing children’s health, having the ‘child reference group’ be children of the same age and gender. Using the Z-score formula, differences are measured between the children and the reference children. The resulting score indicates the extent to which the studied children deviate from the standard population of the same age and gender to reflect the long-term nutritional health of the children. Additionally, researchers believe that children’s height is closely related to long-term nutritional status, psychological development, mortality, and salary level in adulthood. Thus, height is likewise an effective indicator of children’s health and well-being [43, 44]. According to the calculation method recommended by WHO, the current study utilizes the age-specific height of American children as the reference value, using the HAZ score to measure children’s health inequalities. If the HAZ score was found to be negative, it indicates that the observed long-term health status of the children was worse than that of the reference children; when the HAZ score was between − 2 and − 3, it indicates that the children were growth-retarded. This study uses HAZ as a measurement of children’s health inequalities, applying it as a dependent variable to analyse the moderating effect of fathers’ education level on the health differences between rural children, migrant children, and urban children.

#### Independent and control variables

The independent variables of this study are three types of children according to the migration state of children (child types) and the fathers’ education level. The variable of child type includes urban children, rural children, and migrant children. The variable of fathers’ education level is a continuous variable which was measured by fathers’ years of schooling. The study also generates the interaction term of the fathers’ education level and the child type.

Additionally, many researchers have viewed socioeconomic status as the most significant cause of health inequalities [19, 45, 46]. It is precisely because family socioeconomic status is an important predictor of health inequalities that this study includes this variable as a control variable. However, the choice of economic status measurement will affect the observed health inequalities in children, and differences in health inequalities between countries or at different time points may vary depending on the utilized measurement standard for wealth [47]. The current study uses the subjective assessment of family socioeconomic status as a control variable. Park and Cormier reviewed the influence of siblings on child health and found that the relationship between the number of siblings and childhood obesity persisted over time [23]. Therefore, this study also uses number of siblings as a control variable. Some literature also found that demographic variables such as gender, age, and ethnicity of children also affect children’s health inequalities [44, 47]; thus, this study also uses children’s gender, age, and race as control variables and incorporates them into statistical models. Gender is a dichotomous variable, with males coded as 0 and females coded as 1. The variables of age and number of siblings are continuous variables.

### Statistical methods

This study uses the methods of descriptive statistics and inferential statistics to analyse the distribution of HAZ among migrant, rural, and urban children in order to compare differences in the health status of the three child types. Using HAZ as a dependent variable, multiple linear regression analysis is applied to analyse the influence of fathers’ education level on the health inequalities of the three types of children and to observe fundamental health differences among the three groups. In addition, regression analysis is performed on the male and female children to consider gender differences pertaining to the fathers’ education level on the health of the three child types. In order to analyse the moderating effect of fathers’ education level, this study also examines the marginal effect of the interaction term between child type and fathers’ education on the dependent variable HAZ, and the marginal effect of the interaction term between gender, child type, and fathers’ education on the dependent variable HAZ. We conducted analysis applying sampling weights; all data processing and statistical analysis work were done using the STATA 14.0 software.

## Results

### Descriptive statistical analysis

Table 1 presents the average health scores for groups of children with different characteristics. From Table 1, it can be found that the average value of HAZ scores of urban children (1.064) is higher than that of migrant children (0.779), while the average value of HAZ scores of migrant children (0.779) is higher than that of rural children (0.612). The health differences between the three types of children show a gradient change from large to small. However, the HAZ scores of migrant children are closer to those of rural children and farther away from urban children. In addition, we can also find that male rural children (0.843) have higher HAZ scores than female rural children (0.372), male migrant children (1.068) have higher HAZ scores than female migrant children (0.461), and male urban children (0.409) have higher HAZ scores than female (0.757). The HAZ scores of Han rural children (0.71) are higher than those of minority rural children (− 0.252), those of Han migrant children (0.792) are higher than those of minority migrant children (0.631), and those of Han urban children (1.087) are higher than those of minority urban children (0.778). Children with more siblings have lower HAZ scores than those of children with fewer siblings, and children with good family economic conditions have higher HAZ scores than children with poor family economic conditions.

**Table 1:** HAZ scores of children with different sociodemographic characteristics

		HAZ Score				*P*-Value
		Rural children (*x ± s*)	Migrant children (*x ± s*)	Urban children (*x ± s*)	Overall children (*x ± s*)	
Gender	Male	0.843 ± 1.127	1.068 ± 1.064	1.409 ± 0.996	1.1 ± 1.097	< 0.001^#^
	Female	0.372 ± 0.949	0.461 ± 0.869	0.757 ± 0.861	0.549 ± 0.917	
Age	Year 11–13	0.784 ± 1.009	0.972 ± 0.986	1.164 ± 0.941	0.976 ± 0.992	< 0.001^#^
	Year 14–18	0.039 ± 1.066	0.401 ± 0.985	0.578 ± 1.035	0.299 ± 1.061	
Race	Han	0.71 ± 1.035	0.792 ± 1.022	1.087 ± 0.961	0.878 ± 1.018	< 0.001^#^
	Minority	−0.252 ± 0.979	0.631 ± 1.011	0.778 ± 1.189	0.243 ± 1.169	
Number of sibling	1 sibling	1.003 ± 0.988	1.056 ± 1.002	1.185 ± 0.925	1.122 ± 0.954	
	2 siblings	0.573 ± 1.041	0.735 ± 1.001	0.863 ± 1.038	0.677 ± 1.039	< 0.001^+^
	3 siblings or above	0.13 ± 1.067	0.417 ± 0.981	0.532 ± 1.064	0.276 ± 1.057	
Family economic condition	Poverty	0.371 ± 1.109	0.612 ± 1.052	0.757 ± 1.116	0.495 ± 1.114	
	Middle income	0.708 ± 1.033	0.789 ± 1.007	1.087 ± 0.954	0.889 ± 1.009	< 0.001^+^
	Affluent	1.02 ± 0.962	1.113 ± 1.015	1.286 ± 0.939	1.186 ± 0.965	
Total		0.612 ± 1.069	0.779 ± 1.022	1.064 ± 0.982	0.823 ± 1.047	

Note: ^#^ Independent Samples T-Test was used, ^+^ F-test was used

### Multivariate linear regression analysis

In Table 2, this study took the HAZ score as the dependent variable and established four statistical models to further analyse the differences in the health status of the three types of children. Model 1 only includes the independent variables of child type and fathers’ education level, while Model 2 incorporates the control variables. Based on Model 2, Model 3 and Model 4 further incorporate the interaction items. According to Table 2, from Model 1 to Model 4, the F-statistic is all significant, while R-square rose from 0.076 to 0.264, indicating that Model 4 explained the variation of 26.4% of the dependent variable.

**Table 2:** Results of multiple linear regression analysis (*n* = 7772)

Dependent variable: HAZ	Model 1	Model 2	Model 3	Model 4
	Coefficient (SE)	Coefficient (SE)	Coefficient (SE)	Coefficient (SE)
Intercept:	0.031 (0.037)	4.879*** (0.189)	4.789*** (0.195)	4.694*** (0.197)
Gender: (ref. = male)		−0.612*** (0.021)	−0.612*** (0.021)	−0.399*** (0.073)
Age:		− 0.329*** (0.013)	− 0.328*** (0.013)	− 0.328*** (0.013)
Race: (ref. = Han race)		0.173*** (0.038)	0.167*** (0.039)	0.166*** (0.039)
Number of siblings:		−0.172*** (0.017)	−0.174*** (0.017)	− 0.181*** (0.017)
Family economic conditions: (ref. = Poverty)				
Middle income		0.176*** (0.026)	0.175*** (0.026)	0.174*** (0.026)
Affluent		0.372*** (0.048)	0.371*** (0.048)	0.376*** (0.048)
Child types: (ref. = Rural children)				
Migrant children	0.096** (0.033)	0.153*** (0.029)	0.287** (0.097)	0.289** (0.097)
Urban children	0.233*** (0.028)	0.165*** (0.026)	0.301*** (0.083)	0.294*** (0.083)
Fathers’ education level:	0.072*** (0.004)	0.042*** (0.004)	0.053*** (0.007)	0.059*** (0.008)
Interaction terms between child type and fathers’ years of education: (ref. = Rural children × father’s education level)				
Migrant children × fathers’ education level			−0.018* (0.008)	−0.011 (0.011)
Urban children × fathers’ education level			−0.016† (0.009)	−0.008 (0.009)
Interaction terms between gender, child type and fathers’ education level: (ref. = Male × Rural children × Fathers’ education level)				
Female × Rural children × Fathers’ education level				−0.016† (0.009)
Female × Migrant children × Fathers’ education level				−0.025** (0.008)
Female × Urban children × Fathers’ education level				−0.028*** (0.007)
F-statistic	212.15***	304.72***	249.68***	198.97***
DF	3	9	11	14
R^2^	0.076	0.261	0.261	0.264

Note: *** *p* < 0.001, ** *p* < 0.01, * *p* < 0.05, † *p* < 0.1

From Model 2 to Model 4, the control variables of gender, age, number of siblings, and family economic conditions were significant. Female children had poorer health than male children; the more siblings there are, the weaker the children’s health status; and children with good family economic conditions had higher level of health than those with poor family economic conditions. These findings are consistent with the conclusions of previous studies [8, 45], demonstrating that the children’s health level varies depending on gender, age, number of siblings, and family economic condition.

In terms of the main independent variables in Model 4, after controlling for the other variables, the HAZ scores of migrant children are found to be 0.289 higher than those of children from rural areas, while the urban children’s HAZ scores are 0.294 higher than those of migrant children. This finding is identical to the results of the descriptive analysis—that is, urban children have higher levels of health than migrant children, and migrant children have higher levels of health than rural children. In terms of the effect of fathers’ education level on children’s health, the children’s HAZ score increases by 0.059 units for each additional year of the father’s education.

Model 3 incorporates interactions between children type and fathers’ educational level. We find that for each additional year of the father’s education, migrant children’s health score was 0.018 lower than rural children. Given the same variable, urban children’s health score was 0.016 lower than rural children. In other words, the return on rural children’s health brought by increased fathers’ education level is higher than that of migrant children and urban children, and the increase in fathers’ education level is found to have a greater influence on the improvement in rural children’s health. Therefore, the fathers’ education moderates the health differences between urban children and rural children, helping to reduce children’s health inequalities between urban and rural children. As for the interactions term of gender, children type, and fathers’ education in Model 4, we find that with the increase of the father’s education level, the HAZ score of female migrant children is 0.025 lower than male rural children, and the HAZ score of female urban children is 0.028 lower than male rural children. This shows that the impact of fathers’ education on the health status of the three types of children is gender-specific and gender regulates the relationship between fathers’ education and the health status of three types of children.

### Marginal effect

**Fig. 1:**
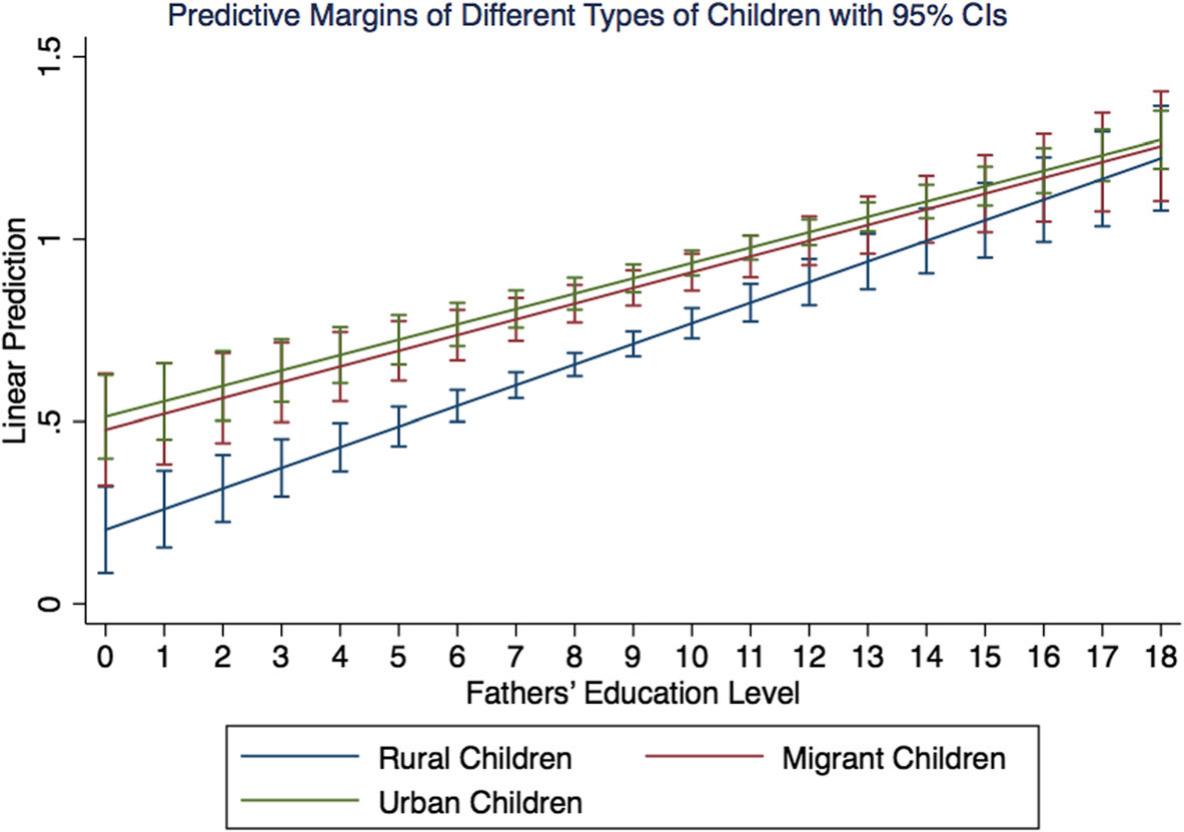
Effects of fathers’ education level on rural, migrant, and urban children’s health

Figure 1 presents the moderating effects of fathers’ education on health differences among the three types of children. This marginal effect is calculated based on Model 3, which includes all the covariates and the interaction term between child type and fathers’ education level. We find that the fathers’ education level has a significant moderating effect on the health differences between urban and rural children. That is, as the education level of the fathers increases, the health inequalities between urban and rural children decrease, indicating that the education level of fathers is crucial to narrowing the health inequalities of urban and rural children. However, it can also be seen from Fig. 1 that increases in fathers’ education level cannot completely bridge the health gap between urban and rural children. Although the health difference between migrant children and urban children is relatively small, an increase in fathers’ education level cannot eliminate the health differences of these two groups, indicating that there is also a significant difference in health between migrant children and urban children; this may be called a healthy ceiling effect for the group of migrant children. Thus, even as migrant children enter the cities and their fathers’ education level increases, the health disparities between migrant children and urban children still exist. The above findings further indicate that structural differences between urban and rural areas still have important shaping effects on children’s health inequalities, and increased fathers’ education level does not eliminate the impact of structural factors affecting the health of migrant children. Due to the altered living environment brought about by migration, migrant children need to integrate and adapt to the living environment of the new city in all dimensions (e.g., economic, psychological and identity). This difficult and long-term process may offset the children’s health benefits brought about by the improvement of the fathers’ education level. Therefore, it appears that creating a good social integration environment and improving the ability of social integration are key factors in improving children’s health.

**Fig. 2:**
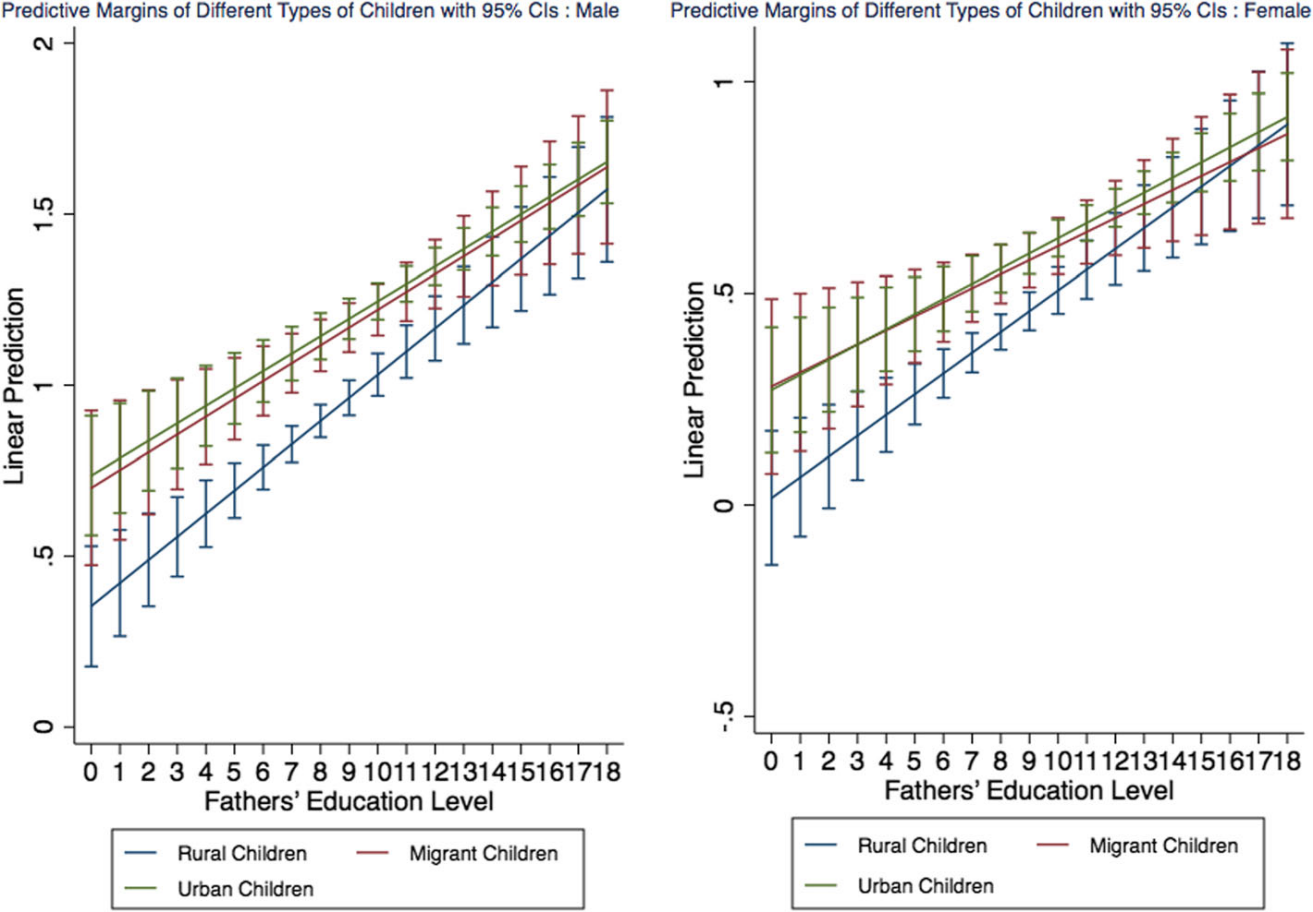
Effects of fathers’ education level on children’s health by gender

Figure 2 shows the marginal impact of fathers’ education on the children’s health inequalities by gender, further analysing the effect of gender in the education level of fathers on the health differences of the three types of children. The marginal effect of Fig. 2 is calculated based on Model 4, which includes all the covariates and interaction variables. It can be seen from Fig. 2 that with increased fathers’ education level, the urban-rural differences in children’s health status still exist with both males and females, but the health differences between migrant children and urban children vary by gender. For male children, the health differences between urban children and rural children gradually narrowed with the improvement of the fathers’ education level, while those between urban children and migrant children decreased slightly, and the health differences between rural children and migrant children likewise reduced. Given an increase in fathers’ education level, the health differences between female urban children and rural children narrowed, but the health differences between female urban children and migrant children increased. These findings indicate that the moderating effect of fathers’ education level on the health inequalities of the three types of children varies by the children’s gender. Overall, we find that the health level of male children and female children vary significantly with a change in the fathers’ education level.

## Discussion

In the context of rural-urban migration, this study explored the health inequalities of urban children, rural children, and migrant children, and the effect of the fathers’ education levels on the health inequalities of these three types of children. While the importance of parental education on children’s health has been studied in many literatures [32, 33, 37], the differential impact of parental education level on urban, rural, and migrant children is still worth discussing. This is of great significance for clarifying the mechanisms of educational achievements of parents on children’s health status in different growth environments, which is caused by macro-structural differences between urban and rural areas.

This study finds that there are significant disparities of health status among the three groups of children, that is, urban children have higher health levels than migrant children, while migrant children have higher health levels than rural children; urban, rural, and migrant children have significant health inequalities. Additionally, the study shows that the fathers’ education level has a significant impact on the children’s health inequalities. The increase in the fathers’ education level can narrow, but not completely bridge, the gap between urban and rural children’s health inequalities. However, increased fathers’ education level does not narrow the health difference between migrant and urban children. Ultimately, we find that the moderating effect of the fathers’ education level on the health inequalities of the three types of children varies with the gender of the child, and the health level of male children and female children varies significantly with a change in the fathers’ education.

Previous studies often focused on the health inequalities of urban and rural children in the framework of urban-rural dual structure, but have not considered and compared the differences of health status among urban, migrant, and rural children. This study finds that the health status of urban children, rural children, and migrant children have a significant gradient effect, wherein urban children have higher health status than migrant children, and migrant children have higher health status than rural children. At the macro level, the health differences may be related to China’s urban-rural dual system and the process of urbanization. Under the urban-rural dual structure system, the health gap between urban and rural areas still exists, and the structural forces represented by the Hukou system still shape the health inequalities of children. Although the shaping role of the Hukou system is weakening, children with urban Hukou still had higher health status (HAZ scores) than those with rural Hukou in China [11]. Meanwhile, the rural population has also shifted to the cities in light of urbanization, resulting in rural left-behind children and migrant children (migrating between rural and urban areas). Existing research also found that the health status of migrant children is better than that of left-behind children [13]. Given the restrictions of the Hukou system, children who move into cities with their parents cannot enjoy the same health benefits as local children; they are disadvantaged in the enjoyment of medical benefits. Therefore, children’s health inequality is not only reflected in the differences in children’s health indicators, but fundamentally, is the result of social structural factors. In China, structural differences between urban and rural areas still have important shaping effects on children’s health inequalities. The urban-rural dual structure represented by the Hukou system and the migration of urban and rural populations caused by the modern urbanization process has become the internal driving force for children’s health inequality.

In terms of the micro-mechanism of the health differences of the three types of children, this study focuses on the impact of fathers’ education on their health. We find that with the increased education level of the fathers, the health gap between urban and rural children shrinks, but does not disappear, and the health differences between migrant children and urban local children have not been eliminated. Although the above findings indicate that the improvement of the fathers’ education level does not completely bridge the huge urban-rural gap between urban and rural children’s health, it does help to reduce the health inequalities between them. The level of education is an important measure of socioeconomic status, which represents the individual’s social class status and the degree of resource possession. The role of the fathers’ education on children’s health level reflects the influence of social determinants of children’s health differences. However, this finding differs from that of Wamani et al., who found that the mother’s education level, rather than father’s education level, was the best predictor of children health inequalities in rural Uganda [32]. These two seemingly contradictory conclusions may be due to differences between the two cultures. In the traditional Chinese Confucian culture system, patriarchy has a great influence, and the father as the leader of the family controls the distribution and decision-making power of family resources. Thus, the role of the father has a more comprehensive and profound impact on the growth and development of the children. On the other hand, the increase in the fathers’ education has not narrowed health differences between migrant and urban children. The findings indicate that, after children enter the cities, increased fathers’ education level does not completely eliminate the structural factors affecting the health of migrant children. This trend may be related to the social integration process of migrants. Some scholars have shown that the problems faced by migrants in the process of social integration result in health differences between urban-rural migrants and urban non-immigrants [48]. Although the health status of migrant children improves with the increase in the economic conditions of migrant children’s families, integration difficulties and institutional constraints of immigrant families in cities, such as the previously mentioned Hukou system, make it difficult to bridge the health disparities between migrant children and urban children.

Furthermore, the moderating effect of fathers’ education level on the three types of children’s health inequality varies with gender. For urban, migrant, and rural children, there is a significant difference in the health levels of male children and female children with increases in fathers’ education level. With the improvement of the fathers’ education level, the difference in health status between urban female children and rural female children decreased, and the degree of reduction is greater than that of males. In other words, the improvement of fathers’ education level can reduce the more urban-rural differences in the health status of female children, relative to male children. As the fathers’ education level increased, the difference in health status between male urban children and male migrant children is slightly decreased, but the difference in health between female urban children and female migrant children is significantly increased. This indicates that there is a gender difference in the health rewards brought by the improvement of the fathers’ education. The health benefits of the fathers’ education level are more likely to manifest in male children, which puts the health status of rural female children and migrant female children at a more disadvantageous position; the health problems of female children are easily ignored.

Several limitations of this study must be considered. First, since this study only uses the cross-sectional data of CEPS 2014–2015, the use of cross-sectional data may have certain limitations in causal inference. Children’s health inequalities may be a process of gradual change, and cross-sectional data does not fully reveal the changing trajectory of children’s health inequalities. Future research can use longitudinal data to describe the trajectory of health inequality of different types of children and reveal its intrinsic evolution mechanism. Second, this study examines the role of fathers’ education on the health status among urban, rural-to-urban migrant children, and rural children, and does not examine the effects of other influential variables such as mothers’ education, although scholars have found that mothers’ education is an important predictor of children’s health inequality [32]. Therefore, if a competitive joint test of the educational level of fathers and mothers is conducted, the results may be more convincing. Third, with regard to the measurement of children’s health, the method of BMI, infant mortality, and self-rated health can all be used to measure children’s health. This study uses the HAZ score which recommend by the WHO to measure children’s health differences. The HAZ score is a standardized indicator that can reflect children’s health inequalities; however, if multiple measurement methods of child health inequalities can be considered simultaneously to test the social causal mechanism of children’s health inequalities, the conclusions may be more effective.

## Conclusion

This study analysed the health inequalities of urban children, rural children, and migrant children, focusing on the impact of fathers’ education level on the health disparities of the three types of children. We found that there are significant health inequalities among the three types of children, and structural differences between urban and rural areas still have important shaping effects on children’s health inequalities. The fathers’ education level is the micro-influence factor of the health inequalities of three types of children. An increase in the fathers’ education level can narrow—but not completely bridge—the gap between urban and rural children’s health inequalities, but not narrow the health differences between migrant and urban children. The effect of the fathers’ education level on the health inequalities of the three types of children varies by the gender of children, with female rural and migrant children having the poorest results, being easily overlooked.

The conclusions of this study are significant for promoting children’s health and formulating children’s health policies. Firstly, special attention should be paid to the health status of rural children in determining child development policies, such as adopting effective child support programs, reducing the dropout rate of rural children—especially rural girls—, and ensuring the adequate nutrition of rural children. Secondly, in the process of reducing health inequalities in children, we must fully consider the children’s socio-ecological environments. The term social ecology refers to the family, school, and community environment in which a child grows up [49]. Socio-ecological environments, including the community in which a child lives and grows, are critical determinants of child growth and development [50]. Therefore, it is necessary to establish a healthy child development environment that covers the family, school, and community, to promote the healthy growth of children by enhancing the living environments, such as improving basic living facilities of families and the learning environment of schools. Thirdly, the social integration of migrant families in the destinations may play an important role in the health promotion of migrant children. It is necessary to improve the health status of migrant children by strengthening the social support of migrant families and creating good social integration environments for this group, as this will likely have positive and long-term effects.

## Data Availability

The datasets used and/or analysed during the current study are available from the National Survey Research Centre at Renmin University of China (NSRC)**,** http://cnsda.ruc.edu.cn.

## Abbreviations

CEPS: China Education Panel Survey
HAZ: Height for Age Z-Scores
PPS: Probability Proportionate to Size Sampling
